# An Investigation of Nurses' Knowledge, Attitudes, and Practices Regarding Disinfection Procedures in Italy

**DOI:** 10.1186/1471-2334-11-148

**Published:** 2011-05-25

**Authors:** Alessandra Sessa, Gabriella Di Giuseppe, Luciana Albano, Italo F Angelillo

**Affiliations:** 1 Department of Experimental Medicine, Second University of Naples, Naples, Italy

## Abstract

**Background:**

This study assessed the level of knowledge, attitudes, and practice regarding disinfection procedures among nurses in Italian hospitals.

**Methods:**

A face-to-face interview gathered the following information: demographic and practice characteristics; knowledge about the healthcare-associated infections (HAIs) and the disinfection practices; attitudes towards the utility of guidelines/protocols and perception of the risks of acquiring/transmitting HAIs; compliance with antisepsis/disinfection procedures; and sources of information.

**Results:**

Only 29% acknowledged that urinary and respiratory tract infections were the two most common HAIs and this knowledge was significantly higher in those with a higher level of education. Attitudes towards the utility of guidelines/protocols for disinfection procedures showed a mean score of 9.1. The results of the linear regression model indicated a more positive attitude in female nurses, in those with a lower number of years of activity, and in those needing additional information about disinfection procedures. Nurses with higher educational level and with a higher perception of risk of transmitting an infectious disease while working were more likely to perform appropriate antisepsis of the surgical wound and handwashing before and after medication.

**Conclusions:**

Plan of successful prevention activities about HAIs and provide pointers to help optimize disinfection procedures and infection prophylaxis and management are needed.

## Background

The issue of healthcare-associated infections (HAIs) continues to be one of the most important public health problems in many countries throughout the world. These infections remain one of the most common complications affecting hospitalized patients and results in morbidity, mortality, and additional costs [1-3]. It is well recognized that the risk of transmission of pathogens when providing medical care and the reduction in the rates of the incidence of HAIs can be kept low through appropriate standardized prevention procedures [4,5]. However, it has been documented that the level of compliance with the use of proven HAIs measures by healthcare workers (HCWs) has been disappointing [6-12], despite the fact that evidence-based procedures promoting appropriate practices in HCWs settings are published [13,14].

It is vital to understand that prevention and control strategies with demonstrated value must be implemented consistently and rigorously. Among the different strategies, the adherence to guidelines for disinfection is an essential ingredient for activities aimed at preventing the HAIs. Accordingly, among the HCWs, nurses have a critical role to play in prevention efforts and they are an important population to study their level of knowledge, attitudes, and behaviour regarding disinfection. However, up to date these issues have received only limited attention [15-20], and obtaining information may be useful for developing programs to increase compliance.

Consequently, the purpose of the present study was to delineate the level of knowledge, attitudes, and appropriate use of disinfection procedures among a random sample of nurses in Italian hospitals and to identify characteristics influencing these outcomes.

## Methods

The cross-sectional study was performed from September 2008 to March 2009 in the geographic area of Caserta and Naples (Italy). The procedures of the study included the following steps: 1) eight non-academic acute general public hospitals were randomly selected; 2) the medical director of each hospital received a letter with the description of the survey and informing of the opportunity to participate, and contact information for study coordinator, so that they could ask questions; 3) from each hospital who agreed to participate, a random sample of working nurses was selected; 4) on the survey date each randomly selected nurse was in person verbally informed by a trained interviewer about the research context, interview procedures, voluntary and anonymous nature of the study, invited to take part, and issued assent form; 5) informed consent or assent to participate in the study was obtained from all the participants; and 6) a face-to-face interview was conducted by trained interviewers, privacy was guaranteed in spite of the nature of the survey, and individual results were kept confidential.

The questionnaire was designed by the authors and included a series of items divided in the following five sections: (1) demographic and practice characteristics; (2) knowledge about the frequency of the HAIs and the disinfection practices; (3) attitudes towards the utility of guidelines/protocols and perception of the risks of acquiring or transmitting HAIs; (4) behaviours and compliance with antisepsis/disinfection procedures; and (5) sources most frequently used to receive up-to-date information about disinfection procedures. The series of answers to the knowledge questions about disinfection practices were arranged by asking respondents to indicate their agreement with true or false statements on a three-point Likert-type scale (ie, agrees, uncertain, disagrees), and about the frequency of the HAIs were as "yes" and "no" choices. Responses to all items assessing attitudes evaluated relating level of agreement or disagreement were on a ten-point Likert-type scale ranging from "1" to "10", meaning "not likely at all" and "very likely" for the two questions on the perceived risk for a HCW to acquire from a patient or to transmit to a patient a HAI and for the question towards the utility of guidelines/protocols for disinfection procedures meaning "not at all" and "very much". Responses to all items assessing the behaviours evaluated whether or not they perform antisepsis/disinfection procedures in their working activity were as "yes" and "no" choices [Additional file 1].

Adherence to guideline recommendations for disinfection was assessed among the respondents by taking into consideration criteria in current use at the time of the survey [17,21-23].

The feasibility of the study and the clarity, quality, and length of the questionnaire items were ensured by means of a pilot-test conducted on a volunteer sample of 20 nurses.

Ethical Committee of the Authors' Institution approved the research protocol, the questionnaire, and the informed consent form.

### Statistical analysis

Multivariate analysis was performed using stepwise logistic and linear regression techniques to evaluate whether individual predictors of interest were independently significantly associated with these three outcomes of interest: knowledge that urinary and respiratory tract infections are the two most common HAIs (Model 1), positive attitudes towards the utility of guidelines/protocols for disinfection procedures (Model 2); performing appropriate antisepsis of the surgical wound and handwashing before and after medication (Model 3). The following explanatory variables were included in all models: gender (male = 0, female = 1), age (continuous, in years), professional role (ordinary nurses = 0, head nurses = 1), educational level (three categories: primary/secondary school = 1, three years registered nurse diploma = 2, baccalaureate/graduate degree = 3), number of years in practice (continuous, in years), ward of employment (medical = 0, surgical = 1), number of hospital beds (continuous), participation in the activities of the Infection Control Committee (no = 0, yes = 1), workshops/seminars and continuing educational courses as sources of information about disinfection procedures (no = 0, yes = 1), and need of additional information about disinfection procedures (no = 0, yes = 1). The following variables were also included: perceived risk of transmitting an infectious disease while working (continuous) in Models 1 and 3; knowledge that non appropriate disinfection increases HCWs' risk of getting and transmitting infectious disease (no = 0, yes = 1) in Model 2; knowledge that surgical wound infections are one of the most frequent HAIs (no = 0, yes = 1), knowledge that non appropriate disinfection increases HCWs workers' risk of transmitting infectious disease (no = 0, yes = 1), perceived risk of getting an infectious disease while working (continuous), and positive attitudes towards the utility of guidelines/protocols for disinfection procedures (continuous) in Model 3.

Predictors of interest found to be related to the outcomes of interest at the univariate level with a *p*-value of 0.25 or better were considered for possible entry in the multivariate linear and logistic regressions equation to assess which factors remained as significant predictors when simultaneously controlling for the rest. For the stepwise analysis, a significance level of 0.2 was used as the criterion for variables to enter the model and 0.4 for variables to remain. Odds ratios (ORs) and their 95% confidence intervals (CIs) were reported as measures of association between predictors and outcomes of interest. All statistical tests were two-sided and *p*-values of 0.05 or less were considered statistically significant. All analysis of the data was conducted using the statistical software Stata 10.0 [24].

## Results

All hospitals reported having a HAIs control committee and they have similar characteristics regarding the availability of qualified HAIs control nurses and of guidelines for hygiene and disinfection procedures. Of the 533 potential respondents approached, a total of 527 subjects consented to be interviewed with a final response rate of 98.9%. The analysis of the demographic and practice characteristics of the study group showed that the majority was female (57.9%), the mean age was 44 years (range 19-67), the mean number of years in practice was 18 (range 6 months-40 years), and more than half worked in surgical wards (53.3%).

Responses to questions about HAIs of the study participants are reported in Table 1. The vast majority of nurses, with frequencies ranging from 77.6% to 96.4%, correctly agreed that the non appropriate application of disinfection procedures increase the risk for a HCW of acquiring/transmitting from/to a patient a HAI. Only less than one third (29%) of the participants acknowledged that urinary and respiratory tract infections were the two most common HAIs. Table 2 reported the results of the multivariate logistic and linear regression models exploring the association between the different variables and the outcomes of interest. Nurses with a higher level of education were more likely to know that urinary and respiratory tract infections were the two HAIs that occurred most frequently (OR = 1.94; 95% CI 1.18-3.19) (Model 1).

**Table 1:** Knowledge about HAIs and disinfection of the 527 nurses who responded to the survey

*Question*						
		
Which of the following are the most common HAIs?	*No.*		*%*			
Urinary tract	302		57.3			
Respiratory	274		52.0			
Surgical wound	261		49.5			
Skin	166		31.5			
Sepsis	143		27.1			
Vascular catheter	142		26.9			
Endocarditis	22		4.2			
Encephalitis	18		3.4			
***Statement***						

	Agree		Uncertain		Disagree	
	***No.***	***%***	***No.***	***%***	***No.***	***%***
Non appropriate disinfection procedures increase the risk of getting HAIs among hospitalized patients	508	96.4	9	1.7	10	1.9
Alcohol-based hand-rubbing should be performed before manipulation of intravenous devices or insertion of an urethral catheters	478	90.7	18	3.4	31	5.9
Disinfectant should be applied for the specified contact time	458	86.9	37	7	32	6.1
Non appropriate disinfection procedures increase the risk of transmitting HAIs among hospitalized patients	424	80.5	31	5.9	72	13.6
Non appropriate disinfection procedures increase the risk of transmitting HAIs among HCWs	410	77.8	53	10.1	64	12.1
Non appropriate disinfection procedures increase the risk of getting HAIs among HCWs	409	77.6	48	9.1	70	13.3

HAIs = Healthcare-associated infections

HCWs = Healthcare workers

**Table 2:** Multivariate logistic (1 and 3) and linear (2) regression models results

Variable	OR	95% CI	*p*
**Model 1**. Knowledge that urinary and respiratory tract infections are the two most common HAIs			
Log likelihood=-311.3, χ^2 ^= 12.35 (4 df), *p *= 0.0195			
Educational level			
Primary/Secondary school*	1.0		
Baccalaureate degree/Graduate degree	1.94	1.18-3.19	0.009
Registered nurse diploma	1.37	0.86-2.19	0.188
Nurses with a higher perception of risk of transmitting an infectious disease while working	1.46	0.97-2.17	0.006
Number of hospital beds	0.99	0.97-1.01	0.164

**Model 3**. Performing appropriate antisepsis of the surgical wound and washing hands before and after medication			

Log likelihood=-260.17, χ^2 ^= 26.31 (7 df), *p *= 0.0004			
Educational level			
Primary/Secondary school*	1.0		
Baccalaureate degree/Graduate degree	1.68	1.06-2.66	0.028
Nurses with a higher perception of risk of transmitting an infectious disease while working	1.56	1.01-2.42	0.049
Nurses working in medical ward	1.4	0.92-2.14	0.115
Head nurses	1.53	0.78-3.01	0.211
Nurses who do not know that non appropriate disinfection increases HCWs' risk of transmitting infectious disease	0.73	0.44-1.21	0.222
Nurses who need additional information about disinfection procedures	1.33	0.75-2.35	0.322

**Variable**	**Coeff.**	***t***	***p***

**Model 2**. Positive attitude towards the utility of guidelines/protocols for disinfection procedures			
F (5,52) = 5.45, *p *= 0.0001, R^2 ^= 4.97%, adjusted R^2 ^= 4.06%			
Female nurses	0.36	2.49	0.013
Nurses with a lower number of years in practice	-0.001	-2.17	0.030
Nurses who need additional information about disinfection procedures	0.38	1.98	0.049
Nurses who know that non appropriate disinfection increases HCWs' risk of getting and transmitting infectious disease	0.28	1.86	0.064
Educational level			
Primary/Secondary school*	1.0		
Baccalaureate degree/Graduate degree	0.3	1.72	0.086
Constant	8.61		

*Reference category

HAIs = Healthcare-associated infections

HCWs = Healthcare workers

Attitudes towards the utility of guidelines/protocols for disinfection procedures, measured on a ten-point Likert scale ranging from 1 to 10 with higher scores indicating more positive attitudes, showed a mean score for the whole sample of 9.1. The results of the linear regression model indicated that a more positive attitude has been observed in female nurses, in those with a lower number of years of activity, and in those needing additional information about disinfection procedures (Model 2 in Table 2). With regard to the aspects of acquiring/transmitting from/to a patient a HAI, respondents overall identified that their main concern was about the risk of acquiring an infection and it was indicated, on scale with a range from 1 to 10, with a mean score of 6.6, whereas a lower score of 4.4 was for transmitting a HAI to a patient.

The vast majority of the nurses self-reported that they perform the disinfection in their working activity. However, among these HCWs, appropriate procedures were observed with different frequencies ranging from 8.1% for placement of urinary catheter to 62.6% for surgical wound disinfection. Particularly, 86.5% made an hand hygiene with antiseptic-containing soap and water or alcohol-based products before invasive procedures (urinary catheter/peripheral venous line insertion), although only 54.9% of them appropriately made an hand hygiene with antiseptic-containing soap and water (59.9%) or alcohol-based products (40.1%). The procedure was not appropriate for 8.7% and 36.4% of nurses who respectively made only an hand hygiene and did not use an appropriate antiseptic product. Moreover, 89.1% made an hand hygiene with antiseptic-containing soap and water or alcohol-based products for the medication of a surgical wound, although only 57.5% of them appropriately made an hand hygiene with antiseptic-containing soap and water (63%) or alcohol-based products (37%), whereas 6.1% washed only the hands and 36.4% did not use the appropriate antiseptic product. Results of the multivariate logistic regression analysis, with the outcome variable of performing appropriate antisepsis of the surgical wound and handwashing before and after medication, revealed that the odds of appropriate behavior were higher if the nurse had a higher educational level (OR = 1.68; 95% CI 1.06-2.66). An appropriate behaviour was more likely in nurses with a higher perception of risk of transmitting an infectious disease while working (OR = 1.56; 95% CI 1.01-2.42) (Model 3 in Table 2).

When presented with a list of various educational sources, approximately three-quarters of all respondents had received information on disinfection procedures. The preferred method for acquiring information was stated to be workshops/seminars and continuing educational courses (71.8%), followed by guidelines/procedures (26.9%), and medical journals (23.2%). The vast majority (82%) of participants would like to improve their level of knowledge.

## Discussion

This survey yielded interesting findings regarding knowledge, attitudes, and disinfection procedures among a random sample of nurses in Italian hospitals.

The data from this study indicated that the current state of nurses' knowledge related to HAIs was poor, particularly is worrying that 29% of the respondents acknowledged that urinary and respiratory tract infections were the two most common HAIs. Analysis of the predictors of being more knowledgeable showed that there was a significant difference in the level of knowledge according to the level of education, because respondents with a university degree were more likely to correctly identify the two HAIs that occurred most frequently compared to those nurses who have a lower level of education. This association may be explained by the fact that those with university curricula were exposed to a higher quality of education and, therefore, they have achieved more information on this topic. In two studies among intensive care nurses, the average knowledge level of evidence-based guidelines for the prevention of ventilator-associated pneumonia was higher in those more experienced [25,26]. In this scenario, HAIs educational programs are not only needed, but also very likely to be welcomed. Workshops/seminars and continuous education programs could provide an accessible source in these topic areas. A high proportion of nurses identified these programs and professional guidelines or standards of practice as preferred source of information. Therefore, hospital administrators should provide support and resources in the form of education and training opportunities designed to increase in this health care personnel the awareness of the disinfection procedures and thereby increase appropriate behaviours with the aim of decreasing HAIs rates. This is also supported by the fact that over three fourths of the study respondents needed additional information regarding disinfection procedures in order to improve their knowledge.

The findings from this survey showed that respondents had an extremely positive attitude towards the utility of guidelines/protocols for disinfection procedures in their activity with a mean score, on a range from 1 to 10, of 9.1. A similar result has been observed in nurses working in operating theatre in Italy with 96.2% that agree that guidelines for disinfection and sterilization practice should be used and maintained [15]. Furthermore, the positive attitude in this sample was independent of the working position and of the knowledge level. Being female, working from a lower number of years, and needing additional information about disinfection procedures were the strongest significant inducements for this group to have a positive attitude.

Nurses surveyed self-reported appropriate disinfection procedures ranging from 8.1% for placement of urinary catheter to 62.6% for surgical wound disinfection. Moreover, actual behaviour before medication was also not always performed in an appropriate manner. Indeed, only 57.5% of those who washed their hands before and after the medication of a surgical wound, appropriately used antiseptic containing soap and water or alcohol-based products and washed hands and wrists. This finding is of great concern since HCWs' hands represent the principal route of transmission of nosocomial pathogens and hands must be decontaminated immediately before each and every episode of direct patient contact/care and after any activity or contact that potentially results in hands becoming contaminated. Moreover, the importance of handwashing programs in preventing and reducing the spread of hospital-associated infections is well recognized [27,28], although a it has been showed that there is still not enough evidence to be certain what strategies improve hand hygiene compliance and multifaceted campaigns with social marketing or staff involvement appear to have an effect [29]. Previous studies have been conducted in order to evaluate handwashing compliance amongst healthcare workers. A higher value has been observed in healthcare workers in Italy with a handwashing compliance of 96.5% for invasive manoeuvres such as urinary catheters [18], whereas, nurses in Turkey commonly washed their hands less frequently than they should and 68.9% of them had a bad quality of handwashing [9]. In the United Kingdom, respectively, only 58.7% and 64.3% nurses, always washed their hands before and after patient contact [30]. It should be noted that nurses with a positive attitude towards the utility of guidelines/protocols for disinfection procedures were more likely to perform appropriate antisepsis of the surgical wound and washing hands before and after medication. More intriguing is the finding that this behaviour did not change significantly with the knowledge.

To appreciate the findings of this current survey, some potential limitations in the design and measurements need to be addressed. First, this is a cross-sectional research design and thus it does not permit analyses of the direction of influence between the different variables and the outcomes of interest. Second, the potential bias attributable to the use of a self-report instrument and we were unable to accurately determine actual behaviours. Direct observations was considered infeasible, as it was expensive and it may also influence behaviour; resulting in exposure estimates that are affected by social desirability. Despite these limitations, this survey resulted in important findings with respect to nurses' knowledge, attitudes, and behaviours regarding disinfection procedures.

## Conclusions

The survey found that the level of knowledge, particularly of the most common HAIs, was not satisfactory and a small percentage of nurses reported that they appropriately perform the disinfection in their working activity. Moreover, the study also revealed an extremely positive attitude towards the utility of guidelines and protocols for disinfection procedures. HAIs control education and training programmes to address these shortfalls and to improve knowledge and adherence to procedures and HAIs prophylaxis and management are essential strategies for patient safety and for the reduction of HAIs.

## Competing interests

The authors declare that they have no competing interests.

## Authors' contributions

AS participated in the design of the study, collected the data, and contributed to the data analysis; GDG participated in the design of the study, collected the data, contributed to the data analysis and interpretation; LA collected the data, contributed to the data analysis and interpretation; IFA, the principal investigator, designed the study, was responsible for the data collection, statistical analysis and interpretation, and wrote the article. The Members of the Collaborative Working Group contributed in recruiting participants. All authors have read and approved the final version of the manuscript.

## Pre-publication history

The pre-publication history for this paper can be accessed here:

http://www.biomedcentral.com/1471-2334/11/148/prepub

## Supplementary Material

Additional file 1 Questionnaire used in the survey Click here for file
