# Supplementary material for: An Investigation of Nurses' Knowledge, Attitudes, and Practices Regarding Disinfection Procedures in Italy
Source: BMC Infect Dis. 2011 May 25;11:148. doi: 10.1186/1471-2334-11-148 (PMC3123570; doi:10.1186/1471-2334-11-148)
Supplement: Additional file 1 — Questionnaire used in the survey [file 1471-2334-11-148-S1.DOCX]

***Questionnaire used in the survey***

**A. DEMOGRAPHIC AND PRACTICE CHARACTERISTICS**

**I will ask you some questions to gather information about your demographic and practice characteristics.**

**A1.** Gender (don’t ask) ⁯ Male ⁯ Female **A2.** How old were you on your last birthday? __________________

**A3.** What is your professional role? ______________ **A4.** What is your highest education level? _____________________

**A5.** How many years have you been working? ______ **A6.** What is your ward of activity? __________________________

**A7.** How many years have you been working in this hospital ward? ____

**A8.** How many hospital beds are there in this ward? ____

**A9.** Is there an Infection Control Committee in your hospital?

□ Do not know □ No □ Yes **A10.** Do/did you participate/participated in the activities of the Committee? □ No □ Yes

**B. KNOWLEDGE**

**I will ask you some questions to explore your knowledge related to healthcare-associated infections (HAIs)**

**B1.** In your opinion, which of the following are the most common HAIs? (more than one answer is allowed)

□ Respiratory □ Infective endocarditis □ Infection of skin lesions **□** Sepsis

□ Urinary tract □ Encephalitis **□** Infection of venous access □ Surgical site

**B2.** For each statement I would like you to answer agree, uncertain, disagree:

**Agree Uncertain Disagree**

1. Disinfectant should be applied for the specified contact time **□**  □ □
2. Non appropriate disinfection procedures increase the risk of

getting HAIs among hospitalized patients **□**  □ □

1. Non appropriate disinfection procedures increase the risk of

transmitting HAIs among hospitalized patients □ □ □

1. Non appropriate disinfection procedures increase the risk of

getting HAIs among healthcare workers (HCWs) □ □ □

1. Non appropriate disinfection procedures increase the risk of

transmitting HAIs among HCWs □ □ □

1. Alcohol-based hand-rubbing should be performed before

manipulation of intravenous devices or insertion of a

urethral catheter □ □ □

**C. ATTITUDES**

**I would like to know your attitudes towards HAIs. Answer the following questions as truthfully as possible.**

**C1.** How do you perceive your risk of getting an infectious disease while working on a 1 to 10 scale with 1 meaning no risk and 10 very much risk?

1 2 3 4 5 6 7 8 9 10

No risk Very much risk

**C2.** How do you perceive your risk of transmitting an infectious disease while working on a 1 to 10 scale with 1 meaning no risk and 10 very much risk?

1 2 3 4 5 6 7 8 9 10

No risk Very much risk

**C3.** How would you rate the utility of the application of guidelines/procedures for disinfection procedures with 1 meaning useless and 10 very useful?

1 2 3 4 5 6 7 8 9 10

Useless Very useful

**D. BEHAVIOURS**

**I am going to ask you some questions which are designed to gather information about your behaviour**

| **1.** Do you perform cleaning procedures before disinfection procedures? □ No □ Yes | | | | |
| --- | --- | --- | --- | --- |
| **2.** Which of the following situation happened during your working activity? | | | | |
|  | **No** | **Yes** | **If the answer is yes, ask:** do you perform skin disinfection  before the procedure? | **If the answer is yes, ask:** do you use guidelines/protocols for this disinfection procedure? |
| Peripheral venous catheterization | □ | □ | □ No □Yes, disinfectant and dilution ___________________ | □ No □Yes, why ____________________________  _____________________________________ |
| Insertion of a urethral catheter | □ | □ | □ No □ Yes, disinfectant and dilution ___________________ | □ No □ Yes, why ____________________________  _____________________________________ |
| Biopsy | □ | □ | □ No □ Yes, disinfectant and dilution ___________________ | □ No □ Yes, why____________________________  _____________________________________ |
| Surgical wound care | □ | □ | □ No □ Yes, disinfectant and dilution ___________________ | □ No □ Yes, why ____________________________  _____________________________________ |
| Intramuscular injection | □ | □ | □ No □ Yes, disinfectant and dilution ___________________ | □ No □ Yes, why ____________________________  _____________________________________ |
| Blood culture collection | □ | □ | □ No □ Yes, disinfectant and dilution ___________________ | □ No □ Yes, why ____________________________  _____________________________________ |
| Intravenous injection | □ | □ | □ No □ Yes, disinfectant and dilution ___________________ | □ No □ Yes, why ____________________________  _____________________________________ |
| Intra-arterial injection | □ | □ | □ No □ Yes, disinfectant and dilution ___________________ | □ No □ Yes, why ____________________________  _____________________________________ |
| Skin contamination with body fluids | □ | □ | □ No □ Yes, disinfectant and dilution ___________________ | □ No □ Yes, why ____________________________  _____________________________________ |
|  | **No** | **Yes** | **If the answer is yes, ask:** do you perform skin disinfection before the procedure? | **If the answer is yes, ask:** do you use guidelines/protocols? |
| Contamination of working surfaces with body fluids | □ | □ | □No □Yes, disinfectant and dilution ___________________ | □No □Yes, why ____________________________  _____________________________________ |
| **3.1** Do you wash your hands before administration of drugs?  □ No **(go to 3.2)** □ Yes, which disinfectant do you use _________________ how long? _______  You wash □ Hands □ Hands and wrists □ Hands, wrist and forarms  Do you use guidelines/protocols? □ Yes □ No, why ___________________________  **3.2** Do you wash your hands before manipulation of intravenous devices or insertion of an urethral catheters?  □ No **(go to 3.3)** □ Yes, which disinfectant do you use _________________ how long _______  You wash □ Hands □ Hands and wrists □ Hands, wrist and forarms  Do you use guidelines/protocols? □ Yes □ No, why ___________________________  **3.3** Do you wash your hands when you perform surgical wound medication?  □ No □ Yes, before medication □ Yes, before and after medication □ Yes, after medication  Which disinfectant do you use _________________ how long _______  You wash □ Hands □ Hands and wrists □ Hands, wrist and forarms  Do you use guidelines/protocols? □ Yes □ No, why ___________________________ | | | | |

**E. INFORMATION**

**I am going to ask you some questions to know the sources and need of information about disinfection**

**E1.** Do you receive information about disinfection?

□ No □ Yes, **if the answer is yes, ask:** From which of the following sources (more than one answer is allowed)

□ Guidelines/procedures □ Workshops/seminars and continuing educational courses □ Colleagues □ Medical journals □ Other____________________

**E2.** In the last year, did you attend any educational course about disinfection in your hospital? □ No □ Yes

**E3.** Do you feel you need more information about disinfection? □ No □ Yes
